# Sex Disparities in Cardiovascular Disease

**DOI:** 10.14797/mdcvj.1328

**Published:** 2024-03-14

**Authors:** Madeline K. Mahowald, Khadeeja Esmail, Fatima M. Ezzeddine, Calvin Choi, Hanna Mieszczanska, Gladys Velarde

**Affiliations:** 1 University of Florida College of Medicine, Jacksonville, Florida, US; 2 Mayo Clinic, Rochester, Minnesota, US; 3 University of Florida College of Medicine, Jacksonville, Florida, US

**Keywords:** women’s cardiovascular health, healthcare disparities, cardiovascular risk factors, ischemic heart disease, treatment gaps, representation in cardiovascular research

## Abstract

Cardiovascular disease is the leading cause of death in women. It remains underdiagnosed, undertreated, and portends worse outcomes in women than men. Disparities exist in every stage of science, from bench research to the editorial board of major journals and in every cardiovascular subspecialty. This review summarizes differences in cardiovascular risk factors and disparities in management and outcomes of ischemic heart disease, heart failure, aortic stenosis, and atrial fibrillation. It also provides an overview of female representation as participants and leaders of clinical trials, editorial boards, and academic institutions. Strategies to overcome these disparities are proposed with examples of successful programs.

## Introduction

Recent strides in understanding the features of cardiovascular disease (CVD) that uniquely or primarily affect women have led to the growth and formalization of the cardio-obstetrics and cardio-oncology fields. This progress reflects the dedicated efforts of providers, scientists, and advocates to improve the care and outcomes of women. Despite decades of recognition, treatment and outcomes, inequalities persist across all disease stages and various cardiovascular subspecialties. Acknowledging the complex interplay between biological (“sex”) and societal (“gender”) factors in CVD and the interchangeable use of these terms in studies, our focus remains on the cumulative effects of individual patient factors, healthcare system factors, public health knowledge gaps, and the limited inclusion of women in research guiding management. This review aims to enumerate these disparities, explore their multifactorial nature, and present examples of successful interventions.

## Risk Factors

CVD is the leading cause of mortality in women globally, with sex-specific differences in the relative risk from traditional and unique risk factors. This section explores these disparities, emphasizing sex-specific and emerging factors in CVD risk alongside the recognition, management, and outcomes within the context of race (Table 1). Managing these risk factors remains the cornerstone of CVD prevention.

**Table 1:** Disparities in cardiovascular risk factors by sex and race. BP: blood pressure; CV: cardiovascular; HDL: high-density lipoprotein; CVD: cardiovascular disease


RISK FACTOR	INCREASES RISK IN MEN/WOMEN	DISPARITIES IN RECOGNITION, MANAGEMENT, AND OUTCOMES	INFLUENCE OF RACE

**Traditional**			

Hypertension	Women (post-menopause)	Women less likely to have hypertension recognized and treated; less BP control	More prevalent and severe in Black women; higher risk of complications

Obesity	Both, higher in women	More stigmatized in women; women respond differently to weight-loss interventions	Black women have the highest rates; disparity less pronounced in men

Diabetes	Higher relative risk in women	Women less likely to achieve care goals; higher CV event risk	Higher incidences in racial minorities; differences in severity of complications

Cigarette smoking	Higher relative risk in women	Harder time quitting for women; more severe CV consequences	Variations in smoking prevalence and cessation rates by race and gender

Dyslipidemia	More atherogenic patterns in women from infancy to early adulthood and middle to old age	Lipid abnormalities less aggressively treated in women	Racial differences in lipid profiles; Black women have higher HDL but also triglycerides

**Nontraditional**			

Autoimmune conditions	More prevalent in women, increasing CVD risk	Women with autoimmune conditions may not receive equal CVD risk assessment	Black women at greater risk due to immunologic and socioeconomic disparities

Prediabetes/metabolic syndrome	Impacts both, higher CVD risk in women	More severe cardiovascular outcomes in women	Higher prevalence and impact in Hispanic and Black women

Cancer treatments	Unique challenges for women with breast cancer and men with prostate cancer	Cardiovascular impact more pronounced in women; increased risk of cardiomyopathy post-anthracycline therapy	Black women at higher CVD risk due to aggressive cancer types and treatment access

Race differences	Higher rates of hypertension and earlier onset of CVD in Black patients	Higher prevalence of hypertension among Black patients; women compounded by delayed diagnosis	Socioeconomic status, healthcare access, and cultural barriers contribute to disparities


### Traditional and Novel Cardiovascular Determinants of Risk: The Role of Sex, Gender, and Socioeconomic Status

The landscape of CVD risk is multifaceted, with sex, gender, race, and socioeconomic status all playing significant roles.

#### Traditional Risk Factors

Cigarette smoking poses a higher relative risk for CVD in women due to metabolic and hormonal differences, and cessation rates are influenced by race and gender, with African American and Hispanic populations often experiencing lower cessation rates.^1,2^

Women face a greater threat from metabolic syndrome, particularly in Hispanic and African American communities where patterns of fat distribution and insulin resistance intensify cardiovascular risks.^3^ Women with diabetes face greater heart disease risks than men, with additional challenges in achieving care goals and a notable disparity in educational resources, especially among racial minorities.^4^ Obesity similarly impacts CVD outcomes in both women and men. The stigmatization and prevalence of obesity varies across genders and races, with Black women having notably higher rates than White or Hispanic women.^5,6^

Hypertension typically develops later in women and is less controlled; it disproportionately impacts Black women, with a high global prevalence and increased risk of complications such as stroke and heart failure.^7,8^

Lastly, compared with men, women typically have a more atherogenic lipid profile from infancy to early adulthood and again post-middle age, but a more favorable profile during premenopause (ages 20 to 50).^9^ The impact of female reproductive stages on lipid profiles and CVD risk remains unclear. Additionally, dyslipidemia in women, influenced by racial disparities, is often undertreated, complicating risk management.^8^

#### Novel Determinants of Cardiovascular Risk

Autoimmune conditions such as lupus and rheumatoid arthritis are more prevalent in women, especially Black women, heightening their CVD risk due to both their medical conditions and socioeconomic challenges that limit healthcare access.^10^

In the realm of cardio-oncology, breast cancer treatment, particularly radiation and chemotherapy, is a primary contributor to elevated CVD risk. This risk is especially pronounced in women, who face a heightened susceptibility. Furthermore, Black women encounter added challenges due to more aggressive cancer types and treatment barriers, underscoring the need for equitable health care.^8,11^

Mental health conditions such as anxiety and depression, which are more common in women, contribute to increased CVD risk.^8^ Gender disparities in recognizing and treating these conditions result in poorer cardiovascular outcomes, particularly for Black women, who often receive inadequate mental health care.^8^

Finally, women are more likely than men to identify expense as a barrier to seeking medical attention.^12^

#### Female-Specific Risk Factors

**Figure 1:**
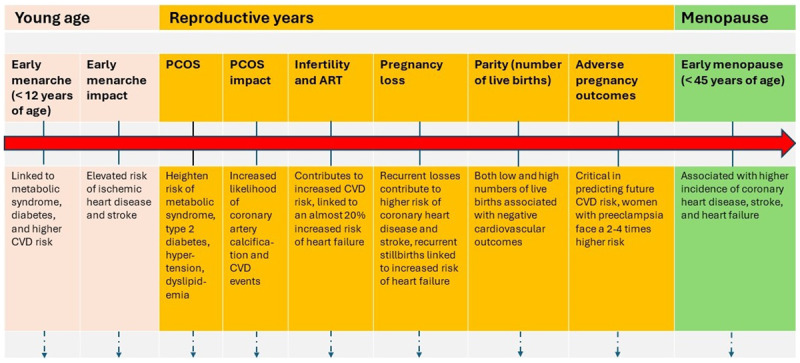
Key female-specific risk factors for cardiovascular disease across a woman’s reproductive lifespan. PCOS: polycystic ovary syndrome; ART: assisted reproductive technology; CVD: cardiovascular disease

Key female-specific risk factors for CVD are deeply interconnected across the reproductive lifespan of women (Figure 1). Early menarche, occurring before the age of 12, is linked to an elevated risk of CVD, metabolic syndrome, and diabetes, whereas a delay in onset translates to a substantial reduction in mortality and lower rates of ischemic heart disease and stroke.^13,14^ Similarly, early menopause, especially before the age of 45, stands as a notable risk factor independently associated with a higher incidence of coronary heart disease, stroke, and heart failure.^13,14^ The mechanism driving this association is thought to be related to the deficiency in protective endogenous hormones. However, it is important to note, based on previous randomized controlled trials (RCTs) and accumulated data, that hormone replacement therapy is currently not recommended for primary or secondary CVD prevention for women of any age.

Polycystic ovary syndrome impacts 5% to 13% of women, heightening the risk of metabolic syndrome, type 2 diabetes, hypertension, and dyslipidemia, and is also associated with a greater likelihood of coronary artery calcification and an increased risk of nearly 30% for CVD events.^15^ Infertility and the use of assisted reproductive technology further contribute to the increased risk of CVD, with infertility itself being linked to an almost 20% heightened risk of heart failure.^16^

The role of spontaneous pregnancy loss is significant, with recurrent losses especially contributing to a higher risk of coronary heart disease and stroke, and recurrent stillbirths specifically linked to an increased risk of heart failure.^13,17^ The number of live births a woman has, known as parity, also presents a complex relationship with CVD risk, with both low and high numbers of live births associated with negative cardiovascular outcomes.^13,18^

Adverse pregnancy outcomes, including hypertensive disorders of pregnancy and gestational diabetes, are critical in predicting future CVD risk, with women who have had preeclampsia facing a risk 2- to 4-times higher, a risk that remains elevated long after pregnancy.^14,19^

In summary, understanding sex differences in CVD risk factors, especially in the context of race and social determinants, is vital for tailoring strategies to the unique needs of women of diverse racial groups. This approach is essential for achieving equitable cardiovascular health.

### Ischemic Heart Disease

Ischemic heart disease in women is underreported and underrecognized.^20^ Despite tremendous improvements in outcomes from ischemic heart disease with contemporary management, gaps remain in the quality of care provided to women and in their outcomes. Distinct anatomy, pathophysiology, and disease pattern are likely contributing factors. However, differences in management of male and female patients in the clinic, emergency department, catheterization lab, cardiac intensive care unit, and hospital wards consistently shortchange women.

#### Acute Coronary Syndromes

The high prevalence, well-established management, and presence of national databases make acute coronary syndromes (ACS) the focus of much research into sex-based disparities in care and outcomes. Biology, patient knowledge, and provider expectations and experience all contribute to disparities (Table 2).

**Table 2:** Factors contributing to worse outcomes in women with acute coronary syndromes. CABG: coronary artery bypass grafting; EKG: electrocardiogram


**Patient factors**

Delay in seeking care Smaller diameter coronary and peripheral arteries Underappreciated nontraditional risk factors Later age and more comorbidities at the time of event Longer hospital stays Higher risk of complications, including access site bleeding

**Provider factors**

Longer door-to-balloon time: delay in getting EKG, delay in seeing provider Less likely to undergo coronary angiography Less likely to receive optimal medical therapy Less likely to receive ideal surgical techniques in CABG, including use of arterial grafts Less likely to be treated with mechanical circulatory support devices

**System factors**

Male-specific reference lab values


Myths persist about women and “atypical” symptoms of chest pain. In fact, at the time of presentation, women and men diagnosed with acute myocardial infarction (MI) are equally likely to report chest pain.^21^ However, women, particularly young women, are more likely to endorse additional symptoms, which may complicate the clinical picture.^21,22^ Women are also more likely to attribute symptoms to alternative causes, including stress, or to perceive that their providers do not suspect cardiac etiology.^21^ Particularly in ST-elevation MI (STEMI), women are more likely to delay seeking medical care after symptom onset.^22,23^ This patient-level delay can be catastrophic; since total ischemic time is a strong predictor of infarct size and clinical outcome, prompt and accurate diagnosis is of paramount importance.^24^ Despite the similarity in presenting complaints, women wait longer to have electrocardiograms and to be seen by emergency providers.^25,26^ Several, but not all, studies additionally report delay in door-to-balloon time, a STEMI metric that is publicly available and sometimes used as a surrogate for “success.”^22,23,26,27^

Women are more likely to present with non-STEMI than with STEMI.^28^ Some studies report higher rates of plaque erosion rather than rupture in women with ACS. However, others have not found a significant difference in plaque composition and inciting event.^29,30^ Regardless of category of ACS, women are less likely to undergo angiography than men, although they receive the same benefit of primary percutaneous coronary intervention (PCI).^31,32^ In fact, Glaser et al. report that women derive equal benefit as men with an early invasive strategy, and that benefit is further enhanced in women with elevated troponin levels.^33^ Women are also less likely to receive optimal medical therapy for MI and for secondary prevention.^27,32^

MI with nonobstructive coronary arteries (MINOCA) is a disease process that is more common in women and has more variation in management. Patients with MINOCA are less likely to receive guideline-directed medical therapy (GDMT) than those with obstructive disease.^34^ Although evidence of benefit is less robust, an observational study showed benefit of statin use and renin-angiotensin-aldosterone system inhibition in this understudied population.^35^ This ambiguity is acknowledged by guidelines, and studies are ongoing to clarify this dilemma.

Due to a multitude of factors, including delays in presentation and intervention, older age, and higher burden of comorbidities, women with ACS consistently show worse in-hospital outcomes than men, including a higher risk of heart failure, cardiogenic shock, and stroke.^28,31,34^ Survival in young women compared with young men is particularly poor irrespective of type of ACS and despite a larger proportion of MINOCA patients, which tend to have a better overall prognosis.^34^

#### Chronic Coronary Syndromes

Symptom burden and anatomic differences are also present in chronic coronary syndromes. Women may have less extensive disease but greater anginal symptoms.^36^ Their coronary arteries are smaller in diameter even when correcting for body surface area, yet intravascular imaging cut-offs for disease severity do not account for differences in body size or sex.^37,38^ When undergoing elective angiogram and PCI, women are more likely to have complications, including access site bleeding and death.^28^ Women are also more likely than men to be diagnosed with ischemia, nonobstructive coronary arteries, and microvascular dysfunction, which incurs symptoms and an increased risk of future major adverse cardiac events, including death, MI, stroke, and hospitalizations for heart failure.^39^

#### Coronary Artery Bypass Grafting

Multiple studies of coronary artery bypass grafting show worse outcomes for women than men both in acute and chronic coronary syndrome settings, including mortality, length of stay, and complications.^31,40^ Indeed, female sex is included as a variable in the validated EuroScore and Society of Thoracic Surgeons score when calculating surgical risk.^41,42^ Women are 14% to 22% less likely to undergo revascularization with optimal surgical technique according to updated guidelines, including anastomosis of the left internal mammary to the left anterior descending artery, multiarterial grafting, and complete revascularization.^40^

#### Strategies to Ensure Equal Care

Although outcomes remain undeniably worse for women, examples exist of successful interventions to narrow the sex gap (Table 3). For example, at one busy quaternary care hospital, the implementation of a 4-facet STEMI protocol that addressed rapid catheterization lab activation and patient transfer, standardized medication administration, and promotion of radial access led to a dramatic decline in the gaps in care between men and women.^27^ Scoring systems such as the HEART, TIMI, and GRACE scores do not include sex or gender as variables and can provide more objective assessments of risk.^43,44,45^ Likewise, standardizing documentation reduced sex disparities in statin prescriptions at discharge in a population of vascular surgery patients.^46^

**Table 3:** Strategies for eliminating disparities in cardiovascular health.


**Patient Factors**

Improve public health messaging around risk and symptoms

**Provider Factors**

Rely on validated scoring systems such as HEART, GRACE, and TIMI to risk stratify and guide management Radial first approach for interventions Recognize comorbidities that heighten risk of cardiovascular disease, such as rheumatologic disease, radiation to the chest, and factors related to pregnancy/fertility and its complications Standardize documentation to ensure optimal medical therapy at the time of discharge

**System Factors**

Utilize sex-specific reference lab values, consider sex-specific end points and analyses in study designs Develop criteria for assessing valvular disease severity that is specific to sex or indexed to body surface area Increase representation of women in clinical trials Identify and address barriers to women’s participation in clinical trials, such as childcare, transportation, financial constraints, language barriers Increase presence of women in academic cardiology settings and journal editorial boards Formalize mentorship programs in academia and research settings


Finally, recognizing the biologic differences between men and women may help tailor our approach to determining risk. Sex-specific troponin values should be implemented, and the presence of large plaque burden or high-risk features on computed tomography may be more predictive of adverse events in women than men.^47,48^

### Heart Failure

Heart failure affects an estimated 2.6 million women in the United States, and there are known multifactorial differences in epidemiology, management, response to treatment, and survival.^49^ Despite the high prevalence, consistent data reveal the suboptimal diagnosis, management, and therapies in women for both heart failure with reduced ejection fraction (HFrEF) and preserved ejection fraction (HFpEF). Patients with HFpEF are more often female and older compared to those with HFrEF, and this accounts for at least half the cases of heart failure in women.^50^

Women are greatly underrepresented in heart failure clinical trials, on average representing 20% to 25% of the cohort. Therefore, the data for current treatment options are based on primarily male-derived data.^51^ One recent exception is the PARAGON HF trial (Prospective comparison of angiotensin receptor neprilysin inhibitor with angiotensin receptor blocker global outcomes in heart failure and preserved left ventricular ejection fraction). This trial recruited more women than in prior trials, and sex was a prespecified subgroup. The trial showed that combination sacubitril/valsartan, compared with valsartan, reduced the likelihood of cardiovascular death and total hospitalizations for heart failure by 27% in women but had no effect in men.^51^ Some reasons for these differences may be the regulation of the constitutive nitric oxide synthases, microvascular inflammation, or dose-response relationships, all of which need further investigation.^51^ In patients with HFrEF, treatment with sacubitril/valsartan is more effective at reducing death and admission to the hospital for heart failure compared with angiotensin converting enzyme inhibitors (ACEIs) and angiotensin receptor blockers (ARBs); this is similar among men and women with systolic dysfunction.^52^

Similarly, clinical trials researching HFrEF are predominantly male, limiting application of data to women. One such example is with GDMT doses. Important gender differences in pharmacokinetics and pharmacodynamics lead to different responses to GDMT.^51^ Pharmacologic studies have shown the maximum plasma concentrations of several ACEIs, ARBs, and beta blockers can be up to 2.5 times higher in women than men despite similar dose administration.^53,54,55^ These findings suggest that no additional benefit may be gained from the target doses currently recommended, and there is a need for gender-based dose targets in HFrEF.^51^ Another example where disparities exist is with regards to gaps in knowledge in peripartum cardiomyopathy and takotsubo cardiomyopathy, which primarily affect women.

#### Device Therapy

Women are less likely to receive implantable cardioverter-defibrillators compared with men; one proposed explanation is less counseling from cardiologists.^56^ Additionally, women’s underrepresentation in RCTs for primary and secondary prevention implantable cardioverter-defibrillator therapy results in these trials being underpowered to assess outcomes in women. Moreover, women are less likely to receive cardiac resynchronization therapy (CRT), despite evidence indicating a higher magnitude of benefit from CRT in women compared to men.^56,57^ Women typically have a shorter mean QRS duration than men. When left bundle branch block develops, the relative increase in QRS and the associated electrical and mechanical dyssynchrony are more pronounced. Furthermore, women are more likely to experience nonischemic etiologies of heart failure, often with lower scar burden, which correlates with more favorable outcomes after CRT implantation.^58^

The use of mechanical support devices is becoming more prevalent in the heart failure population.^59^ However, these devices are underutilized in women, and mortality is higher in women after implantation.^59^ The trends are similar with regard to left ventricular assist devices, with women representing approximately 22% of patients receiving devices in 2016.^60^

Women comprise about 25% of orthotopic heart transplant recipients. Two main reasons for this underrepresentation are selection and referral bias.^61^ Women often receive referrals at later disease stages, which diminish their suitability for transplant. However, when they are referred, they are younger with fewer comorbidities compared to men, and their survival does not differ from men.^61,62^ Additional challenges for women include lack of insurance, absence of caregivers, poor health literacy, and depression.^62^

Overcoming sex and gender disparities in heart failure care is critical to provide equitable treatment to the millions of women affected by this condition.

### Atrial Fibrillation

Women have lower rates of atrial fibrillation (AF) compared with men, although this may be in part explained by lower detection rates.^63^ When women develop AF, they are more likely to have symptomatic AF, heart failure, stroke, and death compared to men.^64,65^ The influence of hormones, patterns of fibrosis, and divergent management strategies may cause some of the apparent differences.

Data on sex differences in atrial electrical and structural remodeling are limited but suggest less shortening in atrial effective refractory period in premenopausal women compared with men and postmenopausal women.^66^ From a structural perspective, female sex has been shown to be a risk factor for atrial fibrosis in patients with AF, which may be the cause of more nonpulmonary vein triggers in women.^67^

In the EURObservational Research Programme-Atrial Fibrillation (EORP-AF) pilot registry, women with symptomatic AF were more likely to be managed with rate control strategies compared to men and were less likely to be treated with catheter ablation.^64^

Women have longer QT intervals at baseline compared to men, which may limit their ability to tolerate antiarrhythmic drugs, particularly class III antiarrhythmic agents. Pregnancy adds another layer of complexity to the management of AF due to the potential teratogenic effects of antiarrhythmic drugs. The 2020 European Society of Cardiology recommends rhythm control in pregnant patients with AF, either via electrical cardioversion or antiarrhythmic drug therapy such as ibutilide or flecainide.^68^

Regarding safety and efficacy of catheter ablation, several studies assessed differences in outcomes based on sex, and results have been divergent. While some studies showed similar safety and efficacy outcomes in both sexes, others showed decreased efficacy and higher risk of adverse events in women.^69,70^ The difference in outcomes was thought to be due to higher rates of persistent AF, nonpulmonary vein triggers, and late referral to ablation.

Female sex is an independent risk factor for stroke in patients with AF. When treated with warfarin for stroke prevention in AF, women have a significantly increased residual risk of stroke or systemic embolism compared with men.^71^ On the other hand, when treated with direct oral anticoagulants, rates of stroke and systemic embolism are similar between men and women.^72^ With regard to left atrial appendage closure, PROTECT-AF and PREVAIL trials showed similar complication rates between men and women.^73,74^

### Aortic Stenosis

Despite a similar prevalence of severe aortic stenosis (AS), baseline characteristics, and risk profile, women are disproportionately less likely to be referred for aortic valve replacement and are referred at later disease stages.^75,76^ The cause of the differential treatment of AS between men and women is multifactorial and encompasses patient, healthcare provider, and healthcare system factors.

At the patient level, pathophysiology and clinical presentation differ between sexes. For example, women tend to have more aortic valve fibrosis than calcification and have different processes of compensatory ventricular remodeling.^77^ Paradoxical low-flow/low-gradient AS is more prevalent in women than men, which may partially account for the underestimation of aortic valve stenosis severity and consequent delays in diagnosis and referral for aortic valve replacement.^77^ These delays likely contribute to worse outcomes in women after aortic valve replacement than in men.

At the healthcare system level, sex-specific references and treatment thresholds have not been established. That is, sex-specific reference values for AS have been proposed, but these have not been formally accepted or translated into changes in the treatment threshold. As such, women are treated at more advanced stages of disease than men.^78^ Despite this delay and a higher risk of procedural complications, women have shown better mortality following transcatheter aortic valve replacement.^79^

A concerted effort at every stage of AS management is required to improve outcomes and mitigate sex-specific disparities. Development of sex-specific reference values, validation in large cohorts, and adaptation and translation of these findings into changes in treatment threshold may be beneficial. Furthermore, public health awareness and educational efforts are required to address misinformation and systemic bias. Artificial intelligence and screening tools could help level the playing field.^80^

### Trial Inclusion

Sex and gender disparities in cardiovascular trial inclusion have been a longstanding concern in the medical community, attracting increased attention in recent years. Despite ongoing efforts to enhance the representation of women in clinical trials, there remains insufficient participation of older patients, women, and individuals from racial minority groups in cardiovascular research.

Basic cell research lays the foundation for understanding diseases, including cardiovascular conditions. Historically, many studies have focused on male cells or animals, neglecting sex differences. This oversight in sex-specific research can result in gaps in our understanding and disparities in treatment outcomes.^81^ Such underrepresentation limits insights into the development and progression of cardiovascular diseases in women and how treatments may affect them differently than in men.

Underrepresentation impacts trials in most subspecialties of cardiology. The recent systematic review of 139 clinical trials with 51,527 participants by Reddy et al. found that over the past 2 decades, women and individuals in racial and ethnic minorities have remained underrepresented in North American valvular heart disease clinical trials, and there have been no significant increases in the representation of older patients, women, and racial and ethnic minority groups over time.^82^ A recent systematic review by Morgan et al. highlighted that heart failure trials consistently have upper age limits and exclude women of childbearing age and those with multiple morbidities.^83^

Using publicly available US Food and Drug Administration (FDA) reviews of trials in support of 36 cardiovascular medications from 2005 to 2015, Scott et al. found large variations in participation of women (range, 22% to 81%; mean per trial, 46%). Women were well represented in trials of drugs for hypertension and atrial fibrillation and underrepresented in trials of heart failure, coronary artery disease, and acute coronary syndromes.^84,85^

A review by Matthews et al. identified several factors affecting womens’ participation in cardiovascular research. Barriers to participation included lack of information and understanding of the research, the women’s lack of awareness of CVD as a significant risk, trial-related procedures, the perceived health status of the participant, and patient-specific factors including travel, caregiving responsibility of women, childcare availability, and cost.^86^

Regulatory agencies and medical organizations have taken steps to promote the inclusion of women in clinical research. For instance, the FDA released guidelines in 2014 encouraging the inclusion of more women in clinical trials.

Enhancing women’s participation in cardiovascular clinical trials is key to understanding sex and gender differences in disease and treatment. This requires raising awareness among researchers and clinicians, educating about gender-specific risks, and mandating womens’ inclusion in trials with sex-specific end points. Overcoming barriers such as childcare and financial constraints is also crucial. These efforts will lead to a more comprehensive understanding of CVD for all genders.

## Leadership, mentorship, authorship, and academia

Cardiology is among the specialties in which women are most underrepresented (21.5% of general cardiology fellows and 12.6% of cardiologists in practice).^87^ This discrepancy extends to academic cardiology, where women account for less than 30% of clinical faculty and less than 20% of senior or leadership positions.^88,89^

Clinical trials change practice in cardiology, and leading them requires research training, mentorship, sponsorship, and networking. A systematic review by Whitelaw et al. demonstrated that among 403 heart failure RCTs published in high-impact medical journals between 2000 and 2019, women comprised only 15.6%, 12.9%, and 11.4% of lead, senior, and corresponding authors, respectively. Among a total of 4,346 authors in any authorship position in these RCTs, 19.6% were women.^90^ Women’s inclusion as authors in major scientific journals increased slightly over recent decades, with some studies showing a narrowing of the gap in first and senior authorship.^91,92,93^

Women are significantly underrepresented on editorial boards and as editors-in-chief compared to men, with women accounting for only 1 in 5 editors-in-chief of leading medical journals.^94,95^ Several factors contribute to these disparities. Historically, the field of cardiology and cardiovascular research has been male dominated. Mentorship is crucial for career development, and the absence of female mentors in senior positions can also hinder the career advancement of female researchers. In fact, papers with women as senior authors were more likely to have women mentees as first authors and to have more diverse research teams.^91^ Formal, structured mentorship programs can successfully address these disadvantages; other male-dominated specialties such as neurosurgery and orthopedics offer successful roadmaps.^88,91^

Implementing blind peer review processes, providing mentorship and networking opportunities for female researchers, advocating for family-friendly policies, and raising awareness about unconscious biases are essential strategies to improve opportunities for women.

## Conclusion

Disparities in women’s cardiovascular health exist in every subspecialty and are multifactorial. A multifaceted approach must encompass inclusion at every level, including patients, providers, researchers, academicians, public health, and system improvements.

## Key Points

- Nontraditional and female-specific risk factors for cardiovascular disease should be incorporated into patient risk stratification and efforts at prevention.
- Women have worse outcomes than men following acute coronary syndrome, which is likely due to patient-specific factors (older age, more comorbidities at the time of presentation) as well as clinical decision making (less likely to undergo coronary angiography, less likely to be treated with appropriate medical therapy).
- Women constitute more than half of the heart failure population but are less likely to be treated with device therapy or undergo heart transplant.
- Formal, structured mentorship programs can combat the underrepresentation of women in academic cardiology, holding leadership positions, on editorial boards, and contributing to scientific research.
